# Chitosan Coagulation Pretreatment to Enhance Ceramic Water Filtration for Household Water Treatment

**DOI:** 10.3390/ijms22189736

**Published:** 2021-09-08

**Authors:** Collin Knox Coleman, Eric Mai, Megan Miller, Shalini Sharma, Clark Williamson, Hemali Oza, Eleanor Holmes, Marie Lamer, Christopher Ly, Jill Stewart, Mark D. Sobsey, Lydia S. Abebe

**Affiliations:** 1 Department of Environmental Sciences and Engineering, Gillings School of Global Public Health, University of North Carolina, Chapel Hill, NC 27599, USA; eric.mai96@gmail.com (E.M.); megmill@live.unc.edu (M.M.); shalinis@live.unc.edu (S.S.); clarkwilliamson09@gmail.com (C.W.); elliebholmes@gmail.com (E.H.); marie.lamer@yahoo.fr (M.L.); chrisly13@gmail.com (C.L.); Jill.Stewart@unc.edu (J.S.); mark_sobsey@unc.edu (M.D.S.); 2 Gangarosa Department of Environmental Health, Rollins School of Public Health, Emory University, Atlanta, GA 30033, USA; hemali.harish.oza@emory.edu; 3 Center for Environment, Energy and Infrastructure, U.S. Agency for International Development (USAID), Washington, DC 20004, USA; lydiashawel@gmail.com

**Keywords:** chitosan, coagulation, flocculation, ceramic filter, filtration, drinking water, household drinking water treatment

## Abstract

Viruses are major contributors to the annual 1.3 million deaths associated with the global burden of diarrheal disease morbidity and mortality. While household-level water treatment technologies reduce diarrheal illness, the majority of filtration technologies are ineffective in removing viruses due to their small size relative to filter pore size. In order to meet the WHO health-based tolerable risk target of 10^−6^ Disability Adjusted Life Years per person per year, a drinking water filter must achieve a 5 Log_10_ virus reduction. Ceramic pot water filters manufactured in developing countries typically achieve less than 1 Log_10_ virus reductions. In order to overcome the shortfall in virus removal efficiency in household water treatment filtration, we (1) evaluated the capacity of chitosan acetate and chitosan lactate, as a cationic coagulant pretreatment combined with ceramic water filtration to remove lab cultured and sewage derived viruses and bacteria in drinking waters, (2) optimized treatment conditions in waters of varying quality and (3) evaluated long-term continuous treatment over a 10-week experiment in surface waters. For each test condition, bacteria and virus concentrations were enumerated by culture methods for influent, controls, and treated effluent after chitosan pretreatment and ceramic water filtration. A > 5 Log_10_ reduction was achieved in treated effluent for *E.coli*, *C. perfringens,* sewage derived *E. coli* and total coliforms, MS2 coliphage, Qβ coliphage, ΦX174 coliphage, and sewage derived F+ and somatic coliphages.

## 1. Introduction

Lack of access to potable water and reliance on surface water contaminated by fecal pollutants as a source of drinking water contributes to high rates of episodic or chronic diarrheal morbidity and mortality. In 2015 it was estimated that 1.3 million deaths annually are associated with diarrheal diseases which disproportionately impact children under 5 years and accounts for 499,000 deaths and 71.59 million Disability Adjusted Life Years (DALYs) [1]. Studies have also associated early childhood diarrheal disease with growth stunting and cognitive impairment [2,3,4]. Viruses have been identified as major contributors to the global burden of diarrheal disease morbidity and mortality with rotavirus alone accounting for 199,000 deaths in 2015 [1,5]. Among viruses, rotavirus is the leading cause of moderate-to-severe diarrhea in infants and children under age 5 and norovirus is most common among adult populations [6,7]. Vaccines exist for rotavirus and have decreased diarrhea-associated morbidity and mortality, but for many waterborne diarrheal pathogens such as noroviruses, *shigella* spp., *campylobacter* spp., *Giardia*, and *Cryptosporidium* there either are no vaccines or their use is not widespread in low and middle income countries [8,9].

Fortunately, low-cost interventions at the household level, such as point-of-use (POU) household water treatment (HWT), can achieve immediate reductions in diarrheal disease and improvements in health outcomes by decreasing exposure to waterborne pathogens [10]. The World Health Organization (WHO) established a scheme for the evaluation of HWT devices utilizing a tiered health-based tolerable risk metric of 10^−6^ and 10^−4^ DALYs per person-year to categorize the efficacy of commercial treatment units. This corresponds to a target log reduction for the following classes of microbes: 4 Log_10_ bacteria, 5 Log_10_ viruses, 4 Log_10_ protozoa for the 10^−6^ DALYs “Highly Protective” tier and 2 Log_10_ bacteria, 3 Log_10_ viruses, 2 Log_10_ protozoa for the 10^−4^ DALYs “Protective” tier, assuming a wastewater content in untreated water of 0.01% by volume [10]. 

POU HWT devices are interim measures for ensuring water safety until access to reliable centrally treated water is available [11]. Single barrier HWT technologies offer simplicity and easy-of-use but have limitations in efficacy of treatment and product longevity. Typical HWT technologies include: filtration systems (ceramic, biosand, cloth), chemical disinfection methods (chlorine, iodine, etc.), coagulation-flocculation, and physical disinfection (heat/boiling and UV radiation). Ceramic water filters (CWF), constructed as described by non-governmental organizations (NGOs) such as Potters without Borders and Potters for Peace, are a widely used HWT and have the advantage of being manufactured locally from clay, grog, and an organic burnout material such as sawdust or rice husk to create pores during the pot firing process [12]. Ceramic water filters treatment efficacy is highly variable by manufacturing location, with reported treatment reductions of 2 to >3 Log_10_ for protozoan parasites and 1 to >4 Log_10_ for bacteria [13,14,15]. Microbial treatment is often further enhanced by the oligodynamic antimicrobial effect of added colloidal silver. However, CWFs effective pore size does not efficiently remove viruses (<1 Log_10_) [16,17,18,19,20]. Van Halem measured effective filter pore size from manufacturers in Cambodia, Ghana, and Nicaragua and found them in the range of 40 µm; and Oyanedel-Craver & Smith observed that 50% of filter pores had diameters ranging from 0.02–15 µm [16,21]. Estimated sizes of *Escherichia coli* and spores of *Clostridium perfringens* are 1–3 µm [22] and fecal indicator viruses are 22.4–28.8 nm for MS2 [23,24], 31.4–33.8 nm for ΦX174 [25], and 21.3–29.4 nm for Qβ [26] coliphages. As both viruses and bacteria are smaller than the effective filter pore size, it is thought that additional removal mechanisms besides physical screening such as sedimentation, diffusion, and adsorption may play a role in microbial retention [16,20,27]. The water treatment process of coagulation-flocculation agglomerates fine particles including microbes, colloidal particles such as clays, and organic matter into larger floc particles which facilitates their removal from water by settling [28]. As many enteric viruses have isoelectric points below the pH of typical drinking waters (pH 6.5–8.5), a cationic coagulant will aid in adsorption of viruses to suspended solids and flocs [29,30,31]. Dual mechanism technologies such as coagulation-flocculation and filtration, or combined coagulation-flocculation and chemical disinfection technologies such as the PUR or Water Maker packets, can address the shortcomings of single barrier treatment and more closely resemble conventional multi-barrier water treatment processes. Combining dual mechanisms of coagulation-flocculation pretreatment before ceramic filtration will result in formation of floc particles large enough to be physically removed from the treated effluent by filtration.

The aim of this study is to examine the use of chitosan, a non-toxic polysaccharide of repeating N-acetyl-D-glucosamine (GlcNAc) and D-glucosamine (GlcN) monomers, as a cationic coagulant pretreatment to aid in the removal of microbial contamination from drinking waters when combined with ceramic filtration. Chitosan is organic, non-toxic to mammals, antimicrobial, and can be functionalized to increase its solubility in water [32]. Conventional drinking water treatment plants utilize inorganic coagulants such as ferric sulfate, ferric chloride, and aluminum sulfate that may not be available in developing countries. Chitosan can be manufactured locally as it is derived from the chitin present in the shells of shrimp and crustaceans, which are abundant in many low resource settings. Local production of chitosan represents a business opportunity for local entrepreneurs and could be supported with training by NGOs currently teaching ceramic filter construction workshops. The cost of chitosan manufacturing will differ by region and availability of feedstocks but was estimated in the USA to be approximately 11.50 $/kg in 2008 [33]. We have previously shown as proof-of-concept that a variety of functionalized chitosans can act as cationic coagulants for POU treatment to remove turbidity and viruses in water over a range of pH, but these studies were limited to ideal lab conditions, synthesized test waters, and short term batch tests, which are not adequately representative of actual conditions where filters are used daily over extended time periods [34,35]. The objectives of this study are to: (1) evaluate the capacity of chitosan acetate (CA) and chitosan lactate (CL) as a cationic coagulant pretreatment combined with ceramic water filtration to remove lab cultured and sewage derived viruses and bacteria in drinking waters, (2) optimize treatment in waters of varying quality and (3) evaluate longer-term continuous treatment over a 10-week daily use period to treat surface waters. To our knowledge, this is the first study to systematically evaluate the long term, repeated use of chitosan as a coagulant pretreatment for ceramic water filtration at the household level using both laboratory strains and environmental microorganism.

## 2. Materials and Methods

### 2.1. Chitosan Preparation and Dosing

Working stocks of 2 g/L chitosan acetate (Heppe Medical Chitosan; degree of deacetylation 80–95%; molecular weight 30–200 kDa) and chitosan lactate (Heppe Medical Chitosan; degree of deacetylation 80–95%; molecular weight 30–500 kDa) were prepared by dissolving chitosan solids in 21 °C autoclaved deionized water and storing at 4 °C. Chitosan lactate and chitosan acetate doses of 10 mg/L and 30 mg/L were diluted from stock solutions for experiments on the initial optimization of microbial removal efficacy and for subsequent sewage containing test waters tested. Using the optimized conditions, a long-term evaluation utilized 10 mg/L of chitosan acetate in surface water collected from an impoundment (see below). Mixing and dosing occurred in open top 5-gallon (19 L) plastic buckets using an 18 in. plastic mixing rod prior to transfer of waters to the ceramic filtration apparatus. Due to the larger volumes used in the long term experiment a rigid plastic container with cover capable of holding ~100 L of water was used. The mixing regimes for coagulation and settling of flocs formed for both initial optimization experiments and then long-term experiments were 30 s rapid mixing (~100 rpm) counter clock wise, followed by 2 min of slow mixing (~25 rpm) clockwise. Mixing was carried out by hand to simulate HWT use conditions.

### 2.2. Ceramic Water Filter

**Figure 1:**
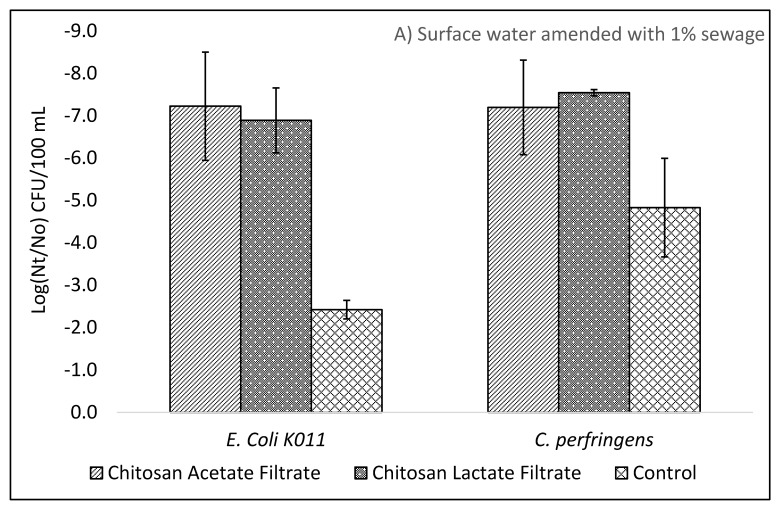

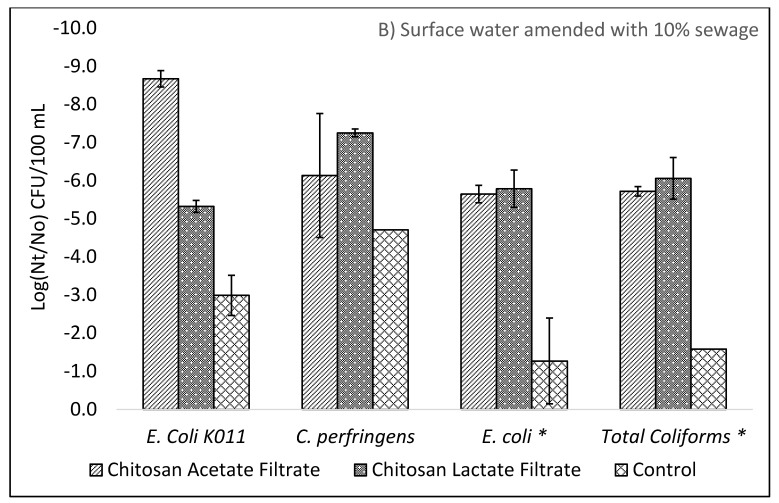
(**A**,**B**) Average bacterial and surrogate protozoa (*C. perfringens* spores) log_10_ reductions after pretreatment with 30 mg/L chitosan acetate or chitosan lactate followed by ceramic filtration for natural surface water amended with (**A**) 1% pasteurized sewage and (**B**) 10% pasteurized sewage (error bars indicate standard deviation). Bacteria with * in (**B**) indicate wild type bacteria derived from unpasteurized sewage.

Ceramic-disc water filters (CWF) as smaller models for ceramic pot filters were manufactured in accordance with the guidelines and materials ratios described in The US Center for Disease Control (CDC) Ceramics Manufacturing Working Group’s Best Practices document [12]. Disc shaped filters of 12 cm diameter were constructed in our UNC lab in Chapel Hill, North Carolina using a custom designed disc mold and hydraulic press (Figure S1), followed by kiln firing by a professional ceramic studio, Claymakers, Durham, NC. Cedar Heights Redart clay (65%), hardwood sawdust (20%), and sand (15%) were measure by weight and dry mixed until homogenous. Deionized water was added incrementally (~30–40% by weight), and mixed to coalesce. Hardwood sawdust was donated from Fitch Lumber Yard in Carrboro, NC and washed quartz sand was purchased at a hardware store in Durham, NC. The volumetric ratio of clay to burnout material was ~1:1 but varied among collections based on the type of lumber the mill processed. All constituents were dried in a drying oven and sieved using a 20/50 mesh screen prior to being weighed and mixed in the stated percentages. Five-hundred-gram quantities of clay mixture were hydraulically pressed into discs (Figure 1), released from the mold, excess trimmed, and allowed to air dry for 72 h. Discs were further dried for 24 h at 70 °C in a drying oven and then kiln fired to cone 010 according to the following schedule: room temperature to 100 °C and hold for 0.5 h at a rate of 50 °C/h; from 100 °C to 600 °C and hold for 0.5 h at a rate of 100 °C/h; from 600 °C to 760 °C and hold for 3 h; and from 760 °C up to 900 °C and hold for 0.5 h at a rate of 100 °C/h to yield a fired finished product approximately 1.5–2 cm thick and 12 cm in diameter. Discs were checked for absences of carbon core to ensure proper burnout, and those that were warped, cracked, or had shrunk in size beyond the desired dimensions were discarded. To evaluate microbial removal and turbidity reduction by chitosan and filtration treatment, silver nanoparticles were not incorporated into the disc filters. Discs were checked for flowrate with deionized water to ensure flow after 1 h exceeded 220 mL/h. For ceramic discs of similar 12 cm diameter and construction this flow rate equates to a minimum ~1.1 L/h for a typical pot shaped filter which falls within manufacturer quality control guidelines of 1–3 L/h for pot filters [12,36].

### 2.3. Test Apparatus Setup

For each test condition, triplicate ceramic disc test filters and one control filter were sealed into rubber pipe couplings with screw clamps and 100% food-safe silicone and allowed to cure for 24–48 h. The pipe couplings were screw clamped to sterilized PVC pipe reservoirs capable of holding 6 L per filter. A single layer of cotton t-shirt pre-filter was attached to each pipe reservoir and each filter apparatus was secured to a wall mount. Sterilized water collection buckets were refreshed daily and filtered effluent volume measured. 

### 2.4. Test Waters

Tests waters consisting of natural waters, or natural waters amended with sewage as recommended by WHO, were selected to represent the range of waters where ceramic filtration technology is utilized [10]. The following test waters were used for chitosan performance evaluations: (1) natural surface water from University Lake in Chapel Hill, NC; (2) lake water amended with 1% pasteurized sewage; (3) lake water amended with 10% pasteurized or unpasteurized sewage; and (4) phosphate-buffered saline as described in Abebe et al., 2016. All lake waters were collected weekly in plastic buckets from a dock extending approximately 50 feet from the lake bank and stored at 4 °C prior to use. For the long-term evaluation, water was collected bi-weekly in 100 L volumes. Raw sewage in the form of primary influent was collected weekly from the Orange County Water and Sewer Authority (OWASA) Wastewater Treatment Plant, in Chapel Hill, NC, transported in coolers, and refrigerated at 4 °C for up to 7 days. Primary effluent was pasteurized the day of experimental test water preparation by heating in a water bath and holding at 70 °C for 30 min. University Lake water parameters as measured by OWASA are reported in Table S2.

### 2.5. Bacteria Detection and Enumeration

The following model bacteria were chosen as indicators of fecal pollution: *Escherichia coli* K011 (ATCC #55124)—a chloramphenicol resistant *E. coli B* mutant and *Clostridium perfringens* (sewage derived spore stock from our UNC lab). *C. perfringens* spores were used as a surrogate protozoan parasite test microbe. *E. coli* stocks were prepared by scraping a loop of frozen *E. coli* into 50 mL Tryptic Soy Broth (TSB Difco) for overnight incubation (18–24 h) at 37 °C on a shaker tray at 100 rpm. Log phase culture was prepared by adding 0.5 mL stationary phase broth culture to 50 mL fresh TSB and incubating at 37 °C for 1.5 h. Log phase culture was aliquoted into 1 mL tubes with 20% glycerol by volume before being stored at −80 °C. Aliquots were mixed with 50 mL of TSB and incubated at 37 °C for 18 to 24 h on days prior to experimental runs. To quantify a desired greater than 6 Log_10_ reduction target after chitosan pretreatment and filtration, *E. coli* was spiked into test waters targeting an initial concentration of 1 × 10^7^ to 1 × 10^8^ CFU/100 mL. *E. coli* K011 was enumerated by spread plate and membrane filtration methods as described in Standard Methods 9215 A and 9215 C on Tryptic Soy Agar (TSA Difco) amended with 34 μg/mL chloramphenicol and incubated for 18 to 24 h at 37 °C. Membrane filtration of 20–100 mL effluent samples was performed with 0.45 μm Millipore membrane filters and incubated for 18 to 24 h at 37 °C. Concentrated wild type *E. coli* and total coliforms stocks were prepared daily by centrifuging 1 L (4 × 250 mL) of sewage at 3000× *g* for 15 min at 4 °C, decanting the supernatant, rinsing with phosphate buffer, and repeating the cycle three additional times before resuspending into a 50 mL final volume. Experiments utilizing sewage derived stocks were assayed by spread plate and membrane filtration on Bio-Rad Rapid E. Coli II agar, with presumptive *E. coli* read as violet colonies (Gal+/Gluc+) and total coliforms as green colonies (Gal+/Gluc−), based on β-D-Glucuronidase (Gluc) and β-D-Galactosidase (Gal) activity, respectively. Sewage derived organisms were not utilized for the long-term experiment due to the inconsistent nature of influent spiking concentrations from each sewage draw and concentration step.

Stocks of *C. perfringens* were prepared by addition of 1 mL *C. perfringens* frozen isolate to 99 mL Modified Duncan Strong (DS) sporulation media and incubated 18–24 h at 44 °C in a Gaspak anaerobic jar system. Stocks of sporulated culture were aliquoted into 1 mL tubes with 20% glycerol by volume before being stored at −80 °C. These aliquots were mixed with 99 mL of DS and incubated at 44 °C for 18 to 20 h on days prior to experimental runs. Due to a goal of quantifying >5 Log_10_ reductions after chitosan pretreatment and filtration, *C. perfringens* was spiked into test waters to yield an initial concentration of 1 × 10^7^ to 1 × 10^8^ CFU/100 mL. *C. perfringens* was enumerated on CP Chromo Select Agar, prepared to manufacturer specifications with D-Cycloserine supplement, by the membrane filtration method described in Standard Methods and 9215 C. Membrane filtration of 20–50 mL effluent samples was performed with 0.45 μm Millipore membrane filters on 60 × 15 mm Petri plates, inverted, and incubated anaerobically for 18 to 20 h at 44 °C in a Gaspak anaerobic jar. After incubation, plates were exposed to ambient aerobic conditions to reveal teal-green colonies counted as *C. perfringens*.

### 2.6. Virus Propagation and Enumeration

The following coliphage viruses were chosen as indicators of fecal pollution: Male specific coliphage (F+) MS2 (ATCC #15597-B1), Q Beta (UNC lab collection initially provided by K. Furuse, Tokai University, Japan) (Stewart 2006), total F+ coliphages propagated from sewage, somatic coliphage ΦX174 (ATCC#13706-B1) and total somatic coliphages propagated from sewage. Male specific coliphages were propagated for 18 to 20 h at 37 °C in TSB broth cultures containing log-phase *E. coli* Famp (ATCC #700609), 0.05 M MgCl_2_ and 15 μg/mL streptomycin and ampicillin on a shaker tray at 100 rpm. Somatic coliphages were propagated for 18 to 20 h at 37 °C in TSB broth cultures containing log-phase *E. coli* CN13 (ATCC #700609), 0.05 M MgCl_2_, and 100 μg/mL nalidixic acid on a shaker tray at 100 rpm. Virus stock was extracted from broth cultures with 10% chloroform by volume and subsequent centrifugation in conical tubes with a swing bucket refrigerated centrifuge at 2600× *g* for 30 min at 4 °C. The supernatant containing the virus stock was allowed to off gas for 30 min prior to being dispensed into 1 mL volumes and frozen at −80 °C. Virus concentration of influent water was enumerated utilizing the Double Agar Layer (DAL) assay and effluent was enumerated using Single Agar Layer (SAL) assay as described in EPA Method 1602. Using host *E. coli* Famp and CN13, respectively, sewage-derived wild-type total F+ and Somatic phages were propagated from OWASA sewage, concentrated by the sloppy-agar method, followed by chloroform extraction and storage at −80 °C.

### 2.7. Physical and Chemical Parameters

Turbidity measurements (NTU) were taken, and percent reductions calculated for pre-filtered influent and post-filtered effluent using a Hach 2100 N Turbidity Meter. The Potential of Hydrogen (pH) was measured using a Denver Instruments Model 215 pH meter with combination electrode. The average flow of filters was measured for control and experimental filters by dividing the filtered volume by the filtration time and reported in mL/h.

### 2.8. Statistical Analysis

Survival fraction (N_t_/N_0_) and Log_10_ reductions (Log_10_(N_t_/N_0_)) were calculated from duplicate plate counts per sample dilution for influent and effluent test waters. Error in reported data, tables, and graphs is expressed as standard deviation. Differences between filtered effluent and filtered effluent pretreated with chitosan were evaluated using nonparametric Mann–Whitney U and Kruskal–Wallis ANOVA with post hoc Dunn’s statistical tests performed in Graphpad Instat 3 and interpreted using a significance level of α = 0.05.

## 3. Results

### 3.1. Optimization Experiments

**Figure 2:**
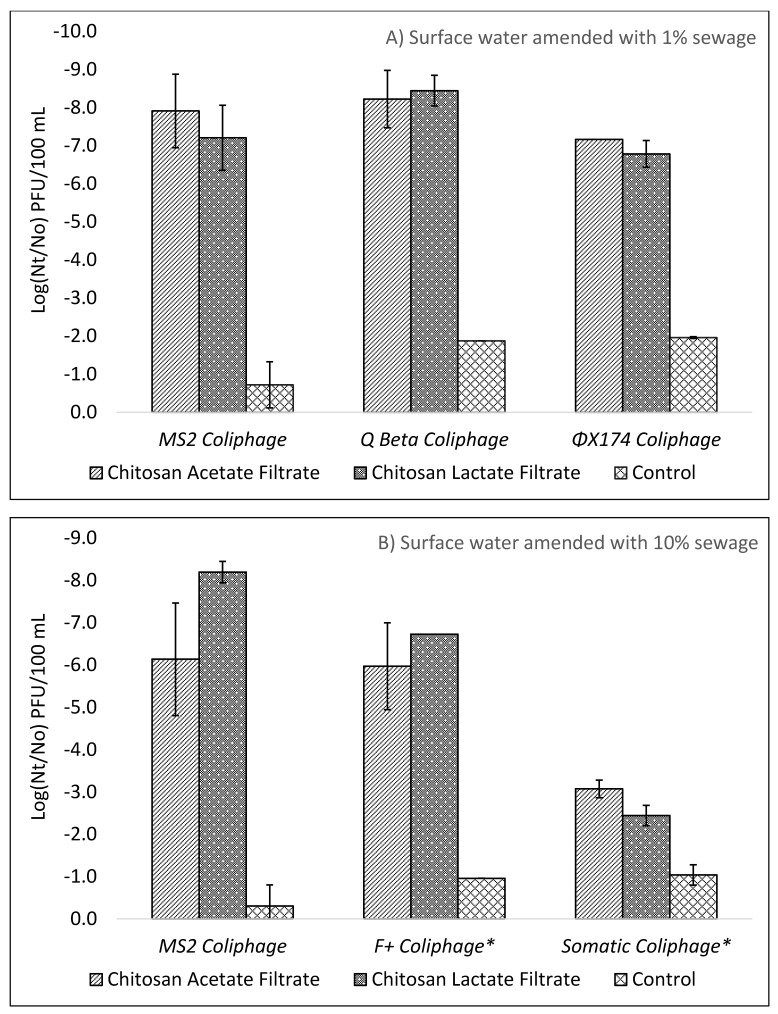
(**A**,**B**) Average virus log reductions after pretreatment with 30 mg/L chitosan acetate or chitosan lactate followed by ceramic filtration for natural surface water amended with (**A**) 1% pasteurized sewage and (**B**) 10% pasteurized sewage (error bars indicate standard deviation). Virus with * in (**B**) indicate wild type total coliphage viruses derived from unpasteurized sewage.

As a prelude to the long-term study, a series of optimization experiments were performed to investigate the impact on treatment efficacy for the choice of test microorganisms, the degree of sewage contamination of test waters, choice of chitosan, and dose of chitosan. As seen in Figure 1 and Figure 2 the addition of either chitosan acetate (CA) or chitosan lactate (CL) as a pretreatment step for ceramic filtered drinking water provided consistently greater reductions of *E. coli*, *C. perfringens,* and coliphage indicator viruses as compared to filtration alone.

### 3.2. Efficacy of Chitosan Pretreatment and Filtration on Bacteria Removal

As shown in Figure 1A,B, filtration without chitosan pretreatment resulted in effluent *E. coli* reductions of 2.3 (±0.78), 2.4 (±0.22), and 3.0 (±0.53) Log_10_ for natural surface water, natural surface water with 1% pasteurized sewage, and natural surface water with 10% pasteurized sewage, respectively. Reduction of *E. coli* in test waters pretreated with 30 mg/L chitosan acetate or lactate were greater than by filtration alone with reductions ranging from 5.3 to 8.7 Log_10_ and exceeding the WHO 3-star performance target for bacteria reduction. In challenge water consisting of a natural surface water amended with 1% pasteurized sewage Log_10_ reductions of *E. coli* were 7.2 (±1.3) and 6.9 (±0.77), respectively, for 30 mg/L chitosan acetate and chitosan lactate doses, which were greater than the 6.2 (±1.05) and 6.2 (±1.05) reductions achieved in the surface water without sewage. No statistically significant difference (*p* < 0.05) was found for *E. coli* reductions between 10 mg/L and 30 mg/L chitosan doses. Therefore, 10 mg/L chitosan acetate dosing was chosen for long-term treatment performance evaluation in surface water. Natural water pretreated with chitosan acetate and chitosan lactate and then filtered resulted in greater reductions of *E. coli* than control filters without pretreatment, regardless of test water or chitosan dose.

Figure 1A,B also present the Log_10_ reductions of spores of *C. perfringens* associated with chitosan pretreatment coagulation, flocculation, and filtration of test waters. *C. perfringens* reductions were 4.6 to 4.8 Log_10_ from filtration alone, which exceeds the WHO HWT “highly protective” performance target of 4 Log_10_ reductions for protozoan pathogens. As shown in Table 1, chitosan pretreatment increased the removal of *C. perfringens* as compared to non-chitosan treated filtrate. Log_10_ reductions from natural waters pretreated with 30 mg/L CA were 5.6 (±0.22) and 7.2 (±1.1) and with 30 mg/L CL they were 6.3 (±1.4) and 7.5 (±0.08) Log_10_ for natural test water and natural test water amended with 1% pasteurized sewage, respectively. No statistically significant difference (*p* < 0.05) was found for *C. perfringens* reductions between chitosan doses of 10 mg/L and 30 mg/L.

### 3.3. Efficacy of Chitosan Pretreatment and Filtration on Virus Removal

In Figure 2A,B are shown the log_10_ reductions of MS2, Qβ, ΦX174, F+, and somatic coliphages from combined chitosan coagulation pretreatment, flocculation, and filtration of a natural test water. Log_10_ reductions for MS2 by filtration without chitosan pretreatment were 0.14 (±0.72), 0.72 (±0.61), and 0.31 (±0.50) in the natural surface water, natural surface water with 1% pasteurized sewage, and natural surface water with 10% pasteurized sewage effluent, respectively. Comparatively, filtered test waters pretreated with chitosan produced greater and statistically significant Log_10_ reductions of 7.7 (±1.1), 7.9 (±0.97), and 6.1 (±1.3) for chitosan acetate and 6.6 (±0.67), 7.2 (±0.85), 8.2 (±0.25) for chitosan lactate in natural surface water, natural surface water with 1% pasteurized sewage, and natural surface water with 10% pasteurized sewage effluent, respectively. In filtered natural waters amended with 1% pasteurized sewage and spiked with coliphage Qβ log_10_ reductions were 8.2 (±0.75) for CA and 8.4 (±0.4) for CL, as compared to 1.9 Log_10_ reductions for filtrate without pretreatment. Somatic coliphage ΦX174 in natural water amended with 1% pasteurized sewage and pretreated with chitosan resulted in 7.2 (±0.0) (CA) and 6.8 (±0.35) (CL) Log_10_ reductions as compared to only 2.0 (±0.02) Log_10_ reductions for ceramic filtrate with no chitosan pretreatment. For MS2 coliphage in phosphate-buffered saline test water Log_10_ reductions were 6.8 (±0.63) (CA) and 8.8 (±0.87) (CL) for effluent pretreated with chitosan and filtered as compared to only 2.0 (±0.45) Log_10_ by filtration only. Overall, reductions of lab strain coliphages in test waters pretreated with chitosan acetate and lactate far exceeded reductions by filtration alone, with reductions ranging from 6.1 to 8.4 Log_10_ and exceeding the 5 Log_10_ WHO “highly protective” criterion.

### 3.4. Removal of Environmentally Derived Microbes

Filtration of surface water amended with 10% raw sewage and spiked with sewage propagated F+ coliphages demonstrated similar reductions of 6.0 and 6.7 Log_10_ by CA and CL pretreatment as compared to laboratory strain MS2 coliphage. However, as seen in Figure 2B, for filtration of natural water amended with 10% raw sewage and spiked with additional sewage propagated somatic coliphages, reductions by CA and CL pretreatment were only 2.4 and 3.1 Log_10,_ respectively_,_ as compared to the >6 Log_10_ reductions seen for lab strain somatic coliphage ΦX174. As shown in Figure 1B, Log_10_ reductions for environmentally derived coliforms from surface water with 10% raw sewage were 5.7 for CA and 6.1 for CL and for *E. coli* they were 5.6 for CA and 5.8 for CL, which exceeded the WHO “highly protective” 4 log_10_ reduction criterion. Overall, environmental strains of *E. coli* and coliforms met WHO reduction standards for both chitosan lactate and chitosan acetate pretreatment as did the lab strain *E. coli*. Reductions were lower by 1.1 Log_10_ for pretreatment with 30 mg/L chitosan lactate and 1.6 Log_10_ lower with 30 mg/L chitosan acetate for environmental *E. coli* in natural water with 10% sewage as compared to lab strain *E. coli* in natural water with 1% sewage. Because bacteria and viruses from environmental sources are not propagated under artificial lab conditions, they provide a more realistic test condition by having greater microorganism diversity that better represents a contaminated source water where ceramic filter technology would be used.

### 3.5. Physical and Chemical Parameters

As seen in Table 1, physical and chemical parameters of turbidity (NTU) and pH were measured for both influent and effluent samples, as was filtration flow rate. Due to variations over time of both natural lake water and collected raw sewage, influent turbidity ranged from 6.0–11.2 NTU for natural water, 4.2–18.4 NTU for natural water with 1% sewage, and 6.9–28.9 NTU for natural water with 10% sewage. Treated water turbidity values and percent reductions associated with chitosan pretreatment and filtration were 1.1 NTU (87.9%) for CA and 0.3 NTU (95.6%) for CL as compared to 4.4 NTU (49.2%) for filtration only from natural water; 2.8 NTU (72.3%) for CA and 2.9 NTU (67.4%) for CL as compared to 3.5 NTU (52.7%) for filtration only from natural water amended with 1% sewage; and 4.2 NTU (40.5%) for CA and 4.3 NTU (70.4%) for CL as compared to 4.9 NTU (55.6%) for filtration only from natural water amended with 10% sewage. The pH of test waters treated with only filtration ranged from 7.1 to 6.8. All test waters pretreated with 30 mg/L chitosan and filtration had 0.1 to 0.4 higher average pH levels than test waters treated with filtration only. Flow rates of surface waters treated with filtration only ranged from 83 to 148 mL/h, while surface waters pretreated with chitosan acetate and filtration ranged from 45 to 101 mL/h, and surface waters pretreated with chitosan lactate and filtration ranged from 39 to 195 mL/h.

### 3.6. Long-term Evaluation of Combined Chitosan Pretreatment and Ceramic Filtration

**Figure 3:**
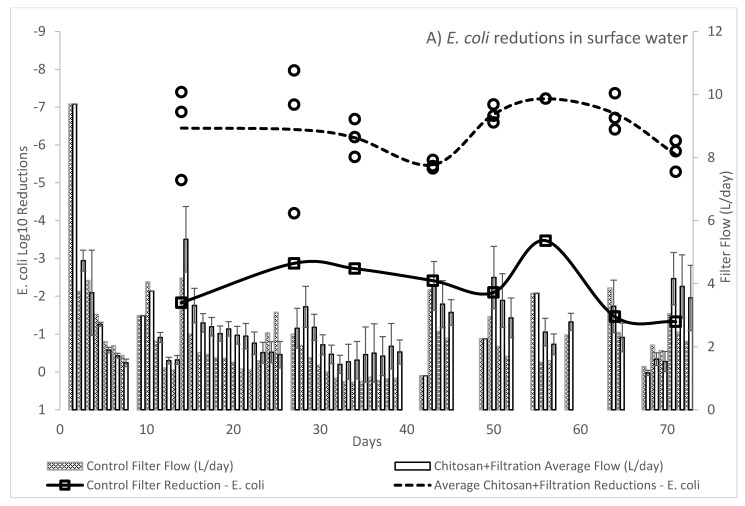

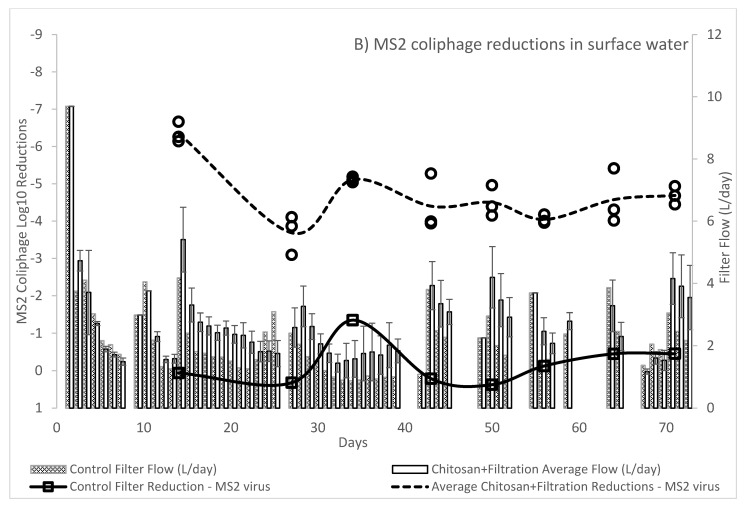
(**A**,**B**) show the Log_10_ reductions of *E. coli* and MS2 coliphage, respectively, by chitosan acetate pretreatment and ceramic filtration (circles) as compared to the control of filtration only(squares). Daily experimental (no-fill) and control (cross-hatch) flow rate (L/day) is displayed as bars. Error bars for flow rate are expressed as standard deviation.

As shown in Figure 3A,B, long term evaluation of a 10 mg/L chitosan acetate dose as a coagulant-flocculant pretreatment prior to ceramic disc filtration exceeded the WHO 4 Log_10_ bacteria reductions target for a three-star “highly-protective” technology and exceeded the WHO 3 Log_10_ two-star “protective” requirement for virus, respectively. After an initial 14-day conditioning period, filter performance was evaluated 8 times for three treatment filters and control filter over a total of 73 days. As seen in Figure 3A, *E. coli K011* average removal was 6.39 (SD: 0.92; *n* = 24) Log_10_ and ranged from 4.19–7.97 Log_10,_ as compared to the control filter Log10 reduction of 2.27 (SD: 0.73; *n* = 8) and ranged from 1.33–3.47 Log_10_. Therefore, the combination of chitosan pretreatment and filtration increased treatment performance for *E. coli* by ~4 Log_10_ or ~99.99% on average as compared to removal by ceramic filtration alone. Removal of culturable MS2 coliphage by chitosan pretreatment and filtration as seen in Figure 3B averaged 4.67 (SD: 0.86; *n* = 24) Log_10_ and ranged from 3.1–6.7 Log_10_ as compared to the control filter Log_10_ reductions of 0.18 (SD: 0.57; *n* = 8) and ranged from −0.32–1.35 Log_10_. The combination of chitosan pretreatment and filtration increased treatment performance for MS2 coliphage by >4 Log_10_ or >99.99% on average, as compared to the removal of MS2 coliphage by ceramic filtration alone.

Over 73 experimental days, 60 daily flow rate measurements were made per filter. As seen in Figure 3, gaps in flow rate data were due to filter disassembly, filter scrubbing, and reassembly of the filter apparatus due to decreased flow (<1.5 L/day), as well as one mandated holiday at day 63. Average flow through the 12 cm diameter ceramic filters across all filters of the chitosan pretreatment group was 2.97 L/day (SD: 1.54; *n* = 180) as compared to 2.52 L/day (SD: 1.54; *n* = 60) for the control filters. Average flow rate of each batch of chitosan pre-treated waters from replicate filters was: Filter A: 2.80 L/day, SD: 1.45; Filter B: 2.33 L/day, SD: 1.27; and Filter C: 2.88 L/day, SD:1.37 (*n* = 60), and exceeded the 2.21 L/day (SD:1.34; *n* = 60) from the control filter. After filter scrubbing, average flow rate declined by 20.6% after 1 day and an additional 12.4% after day 2 from a post scrubbing average of 3.71 L/day (SD: 1.19; *n* = 21) for the test filters and 3.11 L/day (SD: 0.94; *n* = 7) for the control filter which coincided with the flow rate and effluent collected on microbial sampling days. The average pH of the pre-treatment surface water was 7.35 (SD: 0.28), control post-filtration was 7.14 (SD: 0.42), and chitosan-pretreatment and filtration was 7.45 (SD: 0.27). Surface waters had an average initial turbidity of 25.50 NTU (SD: 10.35). Settling of chitosan pre-treated water averaged an 87.3% reduction (3.25 NTU; SD: 1.58)) as compared to a 75.9% reduction (4.63 NTU; SD: 3.37) by settling alone. Combined chitosan-pretreatment and filtration resulted in an average 1.70 NTU (SD: 0.92; 93.3% reduction) and filtration alone resulted in an NTU of 1.76 NTU (SD: 1.32; 93.1% reduction). Only 5 of the 21 turbidity values for chitosan pretreatment and ceramic filtration in the long-term study fell below the WHO drinking water quality recommended < 1 NTU turbidity in a treated drinking water.

## 4. Discussion

The WHO Household Water Treatment and Safe Storage scheme requires that a technology achieve a health-based tolerable risk metric of 10^−6^ or 10^−4^ DALYs by reduction in bacteria, viruses, and protozoa in treated drinking water. The use of chitosan as a coagulant-flocculant pretreatment followed by ceramic filtration resulted in drinking waters exceeding the 10^−4^ DALYs “Protective” treatment requirements as specified in the WHO Evaluating Household Water Treatment Options. Initial optimization experiments utilizing both chitosan acetate and chitosan lactate exceeded the 10^−6^ DALYs “Highly Protective” targets, however the long-term evaluation average virus Log_10_ removal was 4.67 (SD: 0.86; *n* = 24) thus failing to meet the 5 Log_10_ reduction target for “Highly Protective” but meeting the 3 Log_10_ reduction target for “Protective.” While individual filter effluent at times achieved a >5 Log_10_ reduction, the WHO International Scheme to Evaluate Household Water Treatment Devices specifies a mean deviation threshold of 0.2 Log_10_ for treated effluents below the treatment target. Thus, the overall effect of chitosan pretreatment followed by ceramic filtration provides a drinking water with tolerable health risk target between 10^−4^ DALYs to 10^−6^ DALYs. 

In contrast to the WHO, the US Environmental Protection Agency (EPA) POU requirement specifies treated water quality targets of a 6 Log_10_ reduction for bacteria, 4 Log_10_ reduction for virus and 3 Log_10_ reduction for protozoa in the EPA 1987 Guide Standard and Protocol for Testing Microbiological Water Purifiers. The long-term evaluation exceeded the EPA 1987 Guide Standard minimum reductions with average 6.39 log_10_ reduction for *E. coli* KO11 and the afore mentioned viral reduction. Given that protozoa are larger than bacteria, it is assumed that devices operating on removal by size exclusion will meet or exceed the bacteria reductions for protozoa. Treatment with combined chitosan and filtration exceeded the 3 Log_10_ reduction target for *C. perfirngens*, a common surrogate for protozoa. 

Prior studies have reported ceramic filtration without colloidal silver reductions of 1.7 to 3.5 Log_10_ for *E. coli* [21] with silver treated filters achieving up to 6 Log_10_ reductions [14]. Protozoa and protozoan surrogates in preceding studies have shown relatively high rates (>4 Log_10_) of removal by ceramic filtration for protozoa such as *Cryptosporidium parvum* [37] and *Giardia lambia* [37] and a 3.3–4.9 Log_10_ removal of protozoan surrogate *C. perfringens* [16]. In the current study reductions of *E. coli* and *C. perfringens* achieved by chitosan pretreatment and filtration exceeded reductions by >4 Log_10_ and >1 Log_10_, respectively, as compared to filtration alone. Removal of sewage derived *E. coli* and total coliforms exceeded 5 Log_10_ for chitosan pretreatment and filtration but were lower than the log reductions achieved by lab strain *E. coli* KO11. Notably, the control filter for sewage derived *E. coli* achieved 1.72 Log_10_ lower reductions than control filters for *E. coli* KO11. The opposite trend was seen for sewage derived viruses. The diverse mixture of sewage derived viruses resulted in increased removal by ceramic filtration alone when comparing MS2 coliphage to total F+ coliphages in surface waters containing 10% sewage. For chitosan pretreatment and filtration, MS2, Qβ, ΦX174, and F+ coliphages all achieved 5 Log_10_ reductions in the optimization experiments whereas sewage derived total somatic phages failed to achieve a 5 Log_10_ reduction as seen in Figure 2 A,B. Sewage derived organisms were concentrated by centrifugation and washing to remove excess dissolved organics, resulting in a wide diversity of mixed microbial strains that may better represents fecally contaminated surface waters as compared to chemically defined model test waters or surface waters amended with lab strain organisms. In addition to coliphages already present in the 10% sewage test water, additional coliphages, derived from sewage but propagated by a culture method to select for *E. coli* Famp and CN13 host specific strains, were spiked to increase concentrations of culturable influent coliphage viruses.

Previous studies have shown highly variable reduction in viruses by filtration using ceramic water filters. Sobsey et al., 2008 estimated the removal effectiveness of ceramic filters to be 0.5–4 Log_10_ reductions [14]. Lantagne, 2001 found ceramic filters impregnated with colloidal silver, made by Potters for Peace, achieved a 0.09–0.5 Log_10_ reduction for MS2 [37]. Brown et al., 2010 demonstrated a MS2 mean reduction of 1.5 and 1.7 Log_10_ in spiked surface waters for silver amended ceramic water filters and non-silvered filters produced in Cambodia [19]. In a long-term evaluation van Halem et al., 2007 found average MS2 removals between 0.5–1 Log_10_ after 5 weeks and 1–2 Log_10_ at 13 weeks for silver amended ceramic filters manufactured in Cambodia, Ghana, and Nicaragua [16]. Van Halem et al., 2007 also found 1–2 Log_10_ reductions of MS2 by a ceramic filter without silver amendment. The results of Brown and Van Halem stand in contrast to the average 0.18 (SD: 0.57; *n* = 8) Log_10_ reductions found for non-silvered control filters in our long-term evaluation studies. Van Halem indicated that the non-silvered filters did form biofilms after 13 weeks of use, which may explain the increased virus removal. Weekly scrubbing of filters in our study periodically reduced accumulated biofilm growth on the surface of the filters. Virus reductions of > 4 Log_10_ from our study were comparable to previous studies using traditional aluminum and iron coagulants combined with membrane microfiltration [38,39]. Specifically, Matsushita et al., 2005 found 5.1 Log_10_ reductions of Qβ coliphage by combined poly-aluminum chloride coagulation and filtration through a monolithic ceramic membrane with 1.0 μm nominal pore size [40]. 

Virus removal efficacy by ceramic filtration is likely influenced by adsorption of viral particles to suspended solids and the filter element, and the formation of colloids larger than the effective filter pore size, rather than direct screening of suspended viral particles by filter pores. Reported effective pore sizes of 33–52 µm [16], 0.6–3 µm [37], and 0.02–15 µm [21] along with filter pores and cracks ranging from 100–500 µm [21,37] are much larger than 20–40 nm (0.02–0.04 µm) viral particles. Viral removal efficacy by filtration alone is expected be insufficient as influent water will preferentially flow through the larger pore size locations of the filter. Bielefeldt et al., 2010 utilized 0.02 µm diameter carboxylated polystyrene microspheres with isoelectric point 2.2–2.5 and zeta potential −23 to −60 at pH 4–7 as a surrogate for viruses and found 6.5 × 10^8^–2.0 × 10^9^ microspheres/mL in CWF rinse waters following treatment of three batches of spiked waters, indicating detachment from filter pore surfaces. Surface waters with high suspended solids gave increased removal of viruses as compared to the 0.21 and 0.45 Log_10_ MS2 reductions in experiments utilizing de-ionized water [20]. Unlike filters utilized in the optimization experiments, filters utilized in our long-term experiments were pre-conditioned for 14 days prior to filtered effluent microbial sampling. Adsorption sites on long-term filters would therefore likely have had increased loading from microbes and suspended solids present in surface water as compared to filters without break in. 

The current study utilizing chitosan salts as a coagulant-flocculant supports likely virus coagulation by adsorption and related physico-chemical mechanisms, floc formation, and subsequent screening retention by ceramic filters as the primary mechanisms of virus removal. Formation of flocs occur as bacteria and viruses adhere to each other and suspended solids by adsorption of chitosan to surfaces. Cationic polyelectrolytes such as chitosan destabilize suspended colloids and induce floc formation by charge-neutralization and particle bridging [41]. Charge-neutralization is the dominant flocculation mechanism for high charge density polymers while low charge density polymers flocculate by particle bridging [41,42]. The positive charge in chitosan suspensions comes from the protonation of amino groups of GlcN which bind to the negatively charged viral and bacterial particles possessing zeta potentials ranging from −1 to −48 mV [43]. MS2 and Qβ coliphages are similar size but have outer capsid isoelectric points of 3.5–3.9 and 5.0, respectively [44]. Both coliphages saw >7 Log_10_ reductions by chitosan pretreatment and filtration, indicating that the magnitude of net-negative charge at the pH (6.7–7.3) of our test waters for these viruses did not have an impact on reductions. Removal of >6 Log_10_ bacteria and >4 Log_10_ virus in this study indicates that microbes adsorbed to chitosan and the sufficiently large flocs formed facilitated physical screening retention by the pores of the ceramic filter. This result is further supported by the finding of Oza et al., 2021 in which average floc size >150 µm was found by particle size analysis for surface waters treated with chitosan acetate under similar mixing conditions [45].

Differences in virus removals between initial optimization experiments and the long-term experiments also may be attributed to the inherent variation in construction and maintenance of locally made low-tech ceramic filters. Filters were visually inspected and not used when found to have visible cracks, high flow variability, incomplete burnout, warping, and unusual shrinkage. However, internal damage or pockets of non-uniformity are still possible. In-country filter construction makes use of grog, generally from impact milled fired ceramic from filters that fail inspection, to reduce shrinkage during firing. We did not have access to grog and thus supplemented the filters with sand to reduce shrinkage. Our filters were constructed of a mixed sawdust acquired from a local lumber mill, but choice of burnout material plays a significant role in filter pore characteristics that may influence treatment efficacy. Commercial candle and ceramic disc filters manufactured in a properly designed and operated industrial setting have access to precision electric kilns and quality controlled raw materials, such as diatomaceous earth, and are thus able to produce a filter with a more controlled, known and measurable pore size distribution. Brown et al., 2012 found that commercially produced ‘mineral pot’ candle-shaped filters were capable of reducing MS2 by 2–3 Log_10_. In contrast, locally manufactured ceramic filters can be constructed from chemically undefined local clays, a wide variety of burn-out materials, and require training and expertise to wood fire in a kiln without electricity. Production variables of ceramic filters between manufacturers are likely to have a crucial impact on microbial removal efficacy [15].

Furthermore, filters used in the initial optimization experiments did not experience as many scrubbing cycles or extended break-in cycles as the filters utilized in the long-term experiments, which may explain why virus removal efficacy stabilized after 7 weeks of continuous use in long-term experiments. Scrubbing the ceramic filter element with a stiff brush improved flow rate but physically removed some of the surface of the filter. Flow rates for the long-term experiment were measured daily and were strongly influenced by hydraulic head. Testing of filter flow with lake water gave flow rates ranging from 225–330 mL/h after one hour of use, which corresponds to 1.1–2.2 L/h for a pot shaped filter, and is consistent with 1–3 L/h flow rate targets for ceramic pot filter manufacturers [12,36]. Testing of filters with lake water containing up to 10% sewage also met the flow rate criteria but required daily scrubbing to remove particulate loading on the filter surface. Scrubbing was also used to prevent development of a biofilm or “schmutzdecke’ on the surface that would facilitate treatment efficacy, but any subsurface microorganisms within the filter matrix would not have been affected. 

To simulate use in a household setting, a surface water from a local lake was utilized. This is a relatively pristine water source that is on access restricted lands and thus may not be impacted by contamination as severely as some less protected and potentially fecally contaminated source waters in developing countries. Surface waters and raw sewage will differ in composition between collections (Table S2) which may impact chitosan pretreatment and filtration performance. This was evident in the observed variation in turbidity present over the course of the long-term evaluation. WHO recommends turbidity levels of <1 NTU in a treated drinking water which correlates to improved microbial quality and supports effective disinfection by chlorination. WHO also recommends that should <1 NTU not be feasible by a HWT technology then <5 NTU provides for a visual palatability turbidity threshold. While average turbidity reductions by both combined chitosan pretreatment and filtration and treatment by filtration only failed to consistently reduce turbidity <1 NTU, both treatment conditions did reliably achieve <5 NTU. Oza 2019 found that under improved coagulation-flocculation mixing conditions turbidity reductions <1 NTU are possible in surface waters treated with chitosan. Additionally, natural waters amended with up to 10% sewage represents a highly contaminated water, yet displayed a treatment efficacy capable of achieving <5 NTU and >5 Log_10_ *E. coli* reductions indicating a resiliency to source water quality where chlorine-demanding solutes would otherwise prevent adequate disinfection.

## 5. Conclusions

In summary: it was found that coagulation and flocculation of water with either chitosan lactate or chitosan acetate prior to ceramic filtration significantly increased virus and bacteria reductions from surface waters with and without added wastewater, as compared to waters treated by ceramic filtration only. The combined processes of chitosan coagulation and flocculation followed by ceramic filtration improved microbial reductions to achieve WHO Log_10_ microbial reduction performance targets of “Highly Protective’ under some test conditions and at least the “Protective” target under all test conditions over a daily use period of 10 weeks. In contrast, Log_10_ microbial reductions of the same test waters filtered directly without chitosan pre-treatment were far lower. Control filters consistently failed to meet any WHO performance targets for viruses but were able to meet “Protective” performance targets for lab strain *E. coli*. Removal of total coliforms, wild type *E. coli,* and coliphages derived from sewage by chitosan pretreatment and filtration met WHO “protective” targets and may better represent organisms present in contaminated water where HWT devices are used. Control filters consistently performed worse against sewage derived organisms as compared to their lab strain counterparts. The microbial reductions by combined chitosan coagulation-flocculation and ceramic filtration were achieved for as long as 10 weeks of daily filter use, indicating that they are sustainable and do not decline over time. Furthermore, water flow rates of treatment filters consistently met or exceeded flow rates of control filters. Until systems for centralized treatment and distribution of safe drinking water are more widely available, chitosan coagulation-flocculation pretreatment can improve the microbial reduction efficacy of POU-HWT devices, and thus aid in decreasing the morbidity and mortality associated with the global burden of diarrheal disease. We consider chitosan coagulation and flocculation followed by ceramic filtration to be a potentially simple and practical POU/HWT system to achieve effective microbial reductions from drinking water in field use and recommend its further evaluation in real-world low resource settings.

## Figures and Tables

**Table 1:** Microbial reductions and physical parameters by doses of chitosan acetate and chitosan lactate for each test water.

Chitosan Concentration and Filtration Status	Test Water Composition	*C. Perfringens* Log_10_ Reduction	*E. Coli* Log_10_ Reduction	MS2 Log_10_ Reduction	Treated Water Turbidity (NTU) and (% Reduction)	pH	Flow Rate (mL/h)
Filtration (F) Only (No Chitosan)	Natural Water (No Sewage)	−4.6 (±0.85)	−2.3 (±0.78)	−0.14 (±0.72)	4.4 (49.2%)	7.1	149
Filtration (F) Only (No Chitosan)	Natural Water + 1% Sewage	−4.8 (±1.2)	−2.4 (±0.22)	−0.72 (±0.61)	3.5 (52.7%)	6.9	83
Filtration (F) Only (No Chitosan)	Natural Water + 10% Sewage	−4.7 (±--)	−3.0 (±0.53)	−0.31 (±0.50)	4.9 (55.6%)	6.8	135
Filtration (F) Only (No Chitosan)	PBS (No Sewage)	−4.3 (±0.63)	−3.9 (±1.4)	−2.0 (±0.45)	2.3 (78.1%)	7.0	81
**Chitosan Acetate**							
10 mg/L + Filtration	Natural Water (No Sewage)	−6.2 (±0.0)	--	−7.9 (±1.03)	3.5 (56.3%)	6.9	91
10 mg/L + Filtration	Natural Water + 1% Sewage	−6.9 (±0.0)	−5.8 (±2.5)	−8.3 (±1.3)	2.5 (61.8%)	6.8	74
30 mg/L + Filtration	Natural Water (No Sewage)	−5.6 (±0.22)	−6.2 (±1.05)	−7.7 (±1.1)	1.1 (87.9%)	7.3	66
30 mg/L + Filtration	Natural Water + 1% Sewage	−7.2 (±1.1)	−7.2 (±1.3)	−7.9 (±0.97)	2.8 (72.3%)	7.0	45
30 mg/L + Filtration	Natural Water + 10% Sewage	−6.1 (±1.6)	−8.7 (±0.21)	−6.1 (±1.3)	4.2 (40.5%)	7.2	101
30 mg/L + Filtration	PBS (No Sewage)	−4.4 (±1.1)	−2.0 (±1.4)	−6.8 (±0.63)	5.0 (45.6%)	7.1	57
**Chitosan Lactate**							
10 mg/L + Filtration	Natural Water (No Sewage)	−5.8 (±0.0)	−5.6 (±1.5)	−6.6 (±0.85)	4.6 (59.2%)	7.0	68
10 mg/L + Filtration	Natural Water + 1% Sewage	−6.2 (±0.0)	−6.9 (±0.38)	−8.4 (±0.27)	1.5 (86.2%)	6.7	33
30 mg/L + Filtration	Natural Water (No Sewage)	−6.3 (±1.4)	−5.9 (±0.16)	−6.6 (±0.67)	0.3 (95.6%)	7.2	195
30 mg/L + Filtration	Natural Water + 1% Sewage	−7.5 (±0.08)	−6.9 (±0.77)	−7.2 (±0.85)	2.9 (67.4%)	7.0	39
30 mg/L + Filtration	Natural Water + 10% Sewage	−7.3 (±0.10)	−5.3 (±0.16)	−8.2 (±0.25)	4.3 (70.4%)	6.9	50
30 mg/L + Filtration	PBS (No Sewage)	−6.9 (±0.31)	−3.5 (±0.30)	−8.8 (±0.87)	3.3 (77.6%)	6.9	64

## Data Availability

Data available upon request from corresponding author.

## Supplementary Materials

The following are available online at https://www.mdpi.com/article/10.3390/ijms22189736/s1.
