# Supplementary material for: Chitosan Coagulation Pretreatment to Enhance Ceramic Water Filtration for Household Water Treatment
Source: Int J Mol Sci. 2021 Sep 8;22(18):9736. doi: 10.3390/ijms22189736 (PMC8472054; doi:10.3390/ijms22189736)

Supporting Information: Chitosan Coagulation Pretreatment to Enhance Ceramic Water Filtration for Household Water Treatment

Authors: Collin Knox Coleman[1], Eric Mai[1], Megan Miller[1], Shalini Sharma[1], Clark Williamson[1], Hemali Oza[2], Eleanor Holmes[1], Marie Lamer[1], Christopher Ly[1], Jill Stewart[1], and Mark D. Sobsey[1], and Lydia S. Abebe[3]

[1] Department of Environmental Sciences and Engineering, Gillings School of Global Public Health, University of North Carolina, Chapel Hill, NC 27599, USA
[2] Gangarosa Department of Environmental Health, Rollins School of Public Health, Emory University, Atlanta, GA 30033, USA
[3] Center for Environment, Energy and Infrastructure, U.S. Agency for International Development (USAID), 1300 Pennsylvania Avenue, NW Washington, D.C.

*Author to whom correspondence should be addressed; E-Mail: Cwrobert@live.unc.edu

Supporting Information:

Table S1: Statistical significance of chitosan pretreatment and filtration as compared to filtration only for Chitosan Acetate and Chitosan Lactate 30 mg/L doses in a natural surface water amended with 1% sewage

Table S2: Physical and chemical water quality parameters for October through January surface waters collected from University Lake, Carrboro, NC.

Table S3: *E. coli* KO11 log reductions for surface water with chitosan pretreatment and ceramic filtration (CWF), control filter, and control and pre-chitosan experimental influent concentrations.

Table S4: MS2 Coliphage log reductions for surface water with chitosan pretreatment and ceramic filtration (CWF), control filter, and control and pre-chitosan experimental influent concentrations.

Table S5: Turbidity (NTU) reductions for surface water with chitosan pretreatment and ceramic filtration (CWF), control filter, and control and pre-chitosan experimental influent.

Table S6: Microbial log reductions for test waters pretreated with chitosan prior to filtration. Samples were taken from the middle of the water column 30 minutes after chitosan coagulation-flocculation and before test water was passed through CWF.

Figure S1: Ceramic disc molding apparatus consisting of a hydraulic press and aluminum disc mold for the formation of ceramic filter discs

Table S1: Statistical significance of chitosan pretreatment and filtration as compared to filtration only for Chitosan Acetate and Chitosan Lactate 30 mg/L doses in a natural surface water amended with 1% sewage

Test	Comparison	Organism	Sum of Ranks	p-value	Number of Samples (N)
Mann-Whitney U	Natural Water +1% Sewage (30 mg/L Chitosan **Acetate**)	MS2 Coliphage	45	<0.0001	9
	Natural Water +1% Sewage (No Chitosan)		108		8
Mann-Whitney U	Natural Water +1% Sewage (30 mg/L Chitosan **Acetate**)	*C. perfringens*	48	0.0012	9
	Natural Water +1% Sewage (No Chitosan)		88		7
Mann-Whitney U	Natural Water +1% Sewage (30 mg/L Chitosan **Acetate**)	*E. coli*	28	0.0003	7
	Natural Water +1% Sewage (No Chitosan)		92		8
Mann-Whitney U	Natural Water +1% Sewage (30 mg/L Chitosan **Lactate**)	MS2 Coliphage	45	<0.0001	8
	Natural Water +1% Sewage (No Chitosan)		108		9
Mann-Whitney U	Natural Water +1% Sewage (30 mg/L Chitosan **Lactate**)	*C. perfringens*	51	0.0052	9
	Natural Water +1% Sewage (No Chitosan)		85		7
Mann-Whitney U	Natural Water +1% Sewage (30 mg/L Chitosan **Lactate**)	*E. coli*	21	0.0007	6
	Natural Water +1% Sewage (No Chitosan)		84		8

Table S2: Physical and chemical water quality parameters for October through January surface waters collected from University Lake, Carrboro, NC.

Parameter	Average± Std. Dev	Unit	Sample #
Turbidity	25.49±10.35	NTU	n = 9
pH	7.35±0.28	--	n = 9
Orthophosphate as Phosphorus	<0.10±0.00	mg/L	n = 14
UV Absorbance at 254 nm	0.12±0.03	cm-1	n = 8
Dissolved Organic Carbon	4.80±0.28	mg/L	n = 8
Total Organic Carbon	5.93±0.37	mg/L	n = 15
E. coli	26.34±49.59	MPN/100 mL	n = 62
Total Coliform	1596.03±2020.16	MPN/100 mL	n = 62

Table S3: *E. coli* KO11 log reductions for surface water with chitosan pretreatment and ceramic filtration (CWF), control filter, and control and pre-chitosan experimental influent concentrations.

Influent/Effluent Assayed	Day 14	Day 27	Day 34	Day 43	Day 50	Day 56	Day 64	Day 71
Pre-chitosan In (CFU/100 mL)	1.9E+07	7.0E+07	4.8E+06	2.4E+06	1.2E+07	8.4E+06	1.2E+07	2.0E+07
Control Influent (CFU/100 mL)	2.1E+07	7.0E+07	4.8E+06	3.0E+06	2.2E+07	2.2E+07	1.9E+07	2.7E+07
Control CWF (LogNt/N0)	-1.83	-2.87	-2.73	-2.41	-2.10	-3.47	-1.46	-1.33
Effluent CWF - A (LogNt/N0)	-7.40	-4.19	-6.68	-5.60	-6.59	-7.22	-6.71	-5.29
Effluent CWF - B (LogNt/N0)	-5.07	-7.07	-5.68	-5.38	-7.07	-7.22	-7.37	-6.11
Effluent CWF - C (LogNt/N0)	-6.87	-7.97	-6.21	-5.44	-6.77	-7.22	-6.41	-5.83

Table S4: MS2 Coliphage log reductions for surface water with chitosan pretreatment and ceramic filtration (CWF), control filter, and control and pre-chitosan experimental influent concentrations.

Influent/Effluent Assayed	Day 14	Day 27	Day 34	Day 43	Day 50	Day 56	Day 64	Day 71
Pre-chitosan In (PFU/100 mL)	1.0E+08	3.3E+05	7.7E+05	9.4E+06	8.8E+06	2.5E+06	1.7E+07	9.4E+07
Control Influent (PFU/100 mL)	1.4E+08	2.3E+05	4.5E+04	1.3E+05	8.0E+06	7.0E+06	4.1E+08	2.4E+08
Control CWF (LogNt/N0)	0.06	0.32	-1.35	0.21	0.38	-0.13	-0.45	-0.45
Effluent CWF - A (LogNt/N0)	-6.26	-3.10	-5.11	-5.27	-4.96	-4.00	-5.41	-4.94
Effluent CWF - B (LogNt/N0)	-6.66	-3.87	-5.19	-3.99	-4.15	-4.18	-4.31	-4.68
Effluent CWF - C (LogNt/N0)	-6.14	-4.10	-5.04	-3.94	-4.39	-3.96	-4.01	-4.45

Table S5: Turbidity (NTU) reductions for surface water with chitosan pretreatment and ceramic filtration (CWF), control filter, and control and pre-chitosan experimental influent.

Influent/Effluent Assayed	Day 14	Day 27	Day 34	Day 43	Day 50	Day 56	Day 64	Day 71
Pre-chitosan In (NTU)	17.7	17.6	17.8	22.5	16.9	20.9	35.1	44.4
Control Influent (NTU)	11.6	11.7	14.7	35.2	24.1	30.6	31.3	43.5
Control CWF (NTU)	2.7	--	1.83	0.489	1.03	4.46	0.784	1.92
Effluent CWF - A (NTU)	2.65	--	1.16	0.755	0.581	1.34	1.82	2.12
Effluent CWF - B (NTU)	2.11	--	0.553	0.704	0.975	4.47	1.86	1.07
Effluent CWF - C (NTU)	1.99	--	2.31	1.64	1.35	2.93	1.99	3.35

Table S6: Microbial log reductions for test waters pretreated with chitosan prior to filtration. Samples were taken from the middle of the water column 30 minutes after chitosan coagulation-flocculation and before test water was passed through CWF.

**Chitosan Concentration (No Filtration)**	**Test Water Composition**	***C. perfringens* Log$_{10}$ Reduction**	***E. coli* Log$_{10}$ Reduction**	**MS2 Log$_{10}$ Reduction**
**Chitosan Acetate**				
10 mg/L	Natural Water (No Sewage)	--4.67	-3.04	-2.30
10 mg/L	Natural Water + 1% Sewage	-3.81	-2.36	-3.16
30 mg/L	Natural Water (No Sewage)	-3.58	-0.18	-2.28
30 mg/L	Natural Water + 1% Sewage	-4.69	-0.70	-1.34
30 mg/L	Natural Water + 10% Sewage	-3.73	--	-1.30
30 mg/L	PBS (No Sewage)	-0.49	-1.75	-2.78
**Chitosan Lactate**				
10 mg/L	Natural Water (No Sewage)	-4.27	-2.40	-2.51
10 mg/L	Natural Water + 1% Sewage	-3.33	-1.72	-3.93
30 mg/L	Natural Water (No Sewage)	-3.41	-1.40	-2.22
30 mg/L	Natural Water + 1% Sewage	-3.92	-1.99	-1.87
30 mg/L	Natural Water + 10% Sewage	-3.57	-0.78	-2.74
30 mg/L	PBS (No Sewage)	-0.77	-1.90	-3.16

Figure S1: Ceramic disc molding apparatus consisting of a hydraulic press and aluminum disc mold for the formation of ceramic filter discs

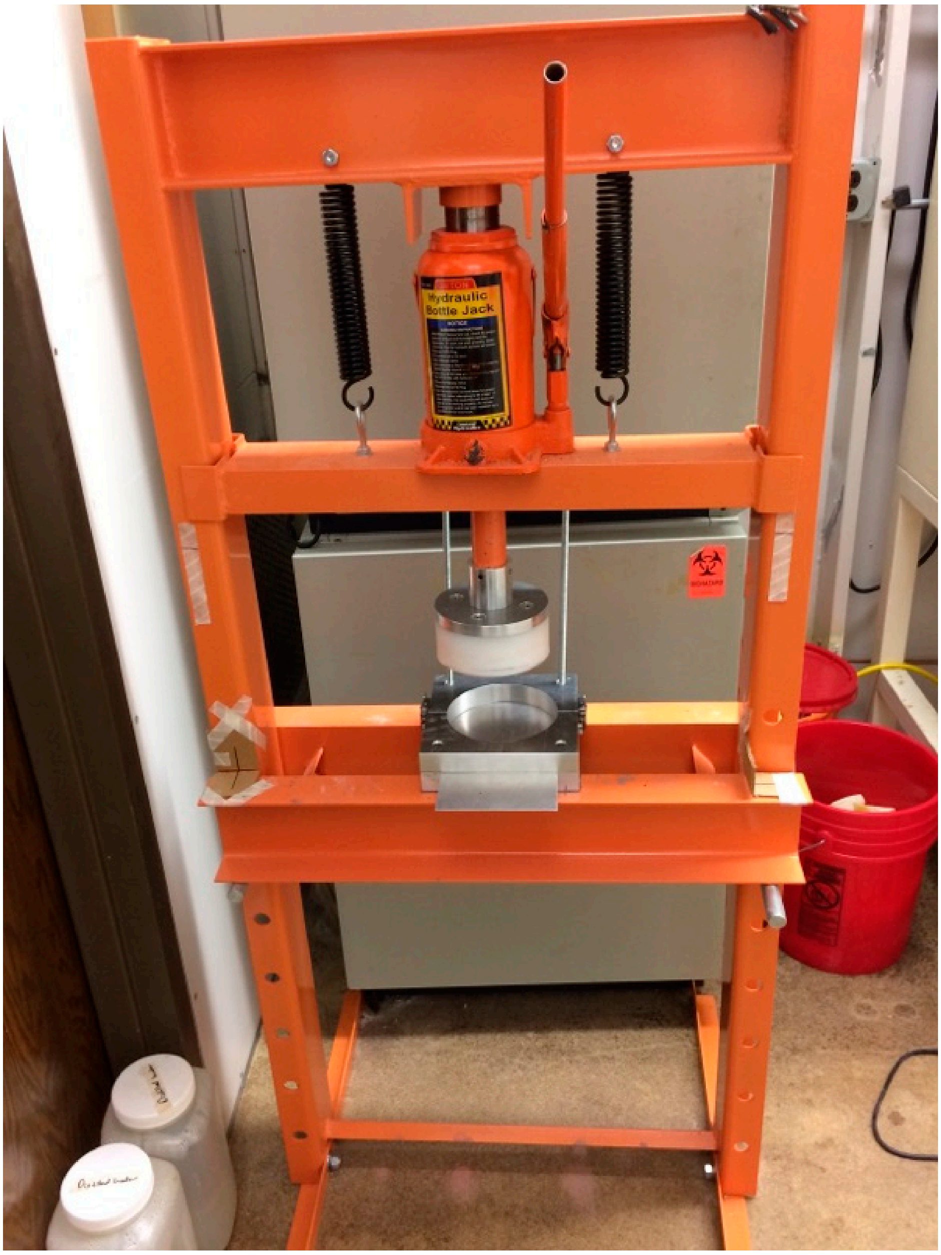

Supplement: Supplementary file 1 [file ijms-22-09736-s001.zip › ijms-1317574-supplementary.pdf]
